# Mitochondrial Signaling Pathways Associated with DNA Damage Responses

**DOI:** 10.3390/ijms24076128

**Published:** 2023-03-24

**Authors:** Tsutomu Shimura

**Affiliations:** Department of Environmental Health, National Institute of Public Health, Wako 351-0197, Saitama, Japan; simura.t.aa@niph.go.jp; Tel.: +81-48-458-6261

**Keywords:** mitochondrial signaling, DNA damage response, oxidative stress, inflammation response, radiation carcinogenesis

## Abstract

Under physiological and stress conditions, mitochondria act as a signaling platform to initiate biological events, establishing communication from the mitochondria to the rest of the cell. Mitochondrial adenosine triphosphate (ATP), reactive oxygen species, cytochrome C, and damage-associated molecular patterns act as messengers in metabolism, oxidative stress response, bystander response, apoptosis, cellular senescence, and inflammation response. In this review paper, the mitochondrial signaling in response to DNA damage was summarized. Mitochondrial clearance via fusion, fission, and mitophagy regulates mitochondrial quality control under oxidative stress conditions. On the other hand, damaged mitochondria release their contents into the cytoplasm and then mediate various signaling pathways. The role of mitochondrial dysfunction in radiation carcinogenesis was discussed, and the recent findings on radiation-induced mitochondrial signaling and radioprotective agents that targeted mitochondria were presented. The analysis of the mitochondrial radiation effect, as hypothesized, is critical in assessing radiation risks to human health.

## 1. Introduction

**Figure 1:**
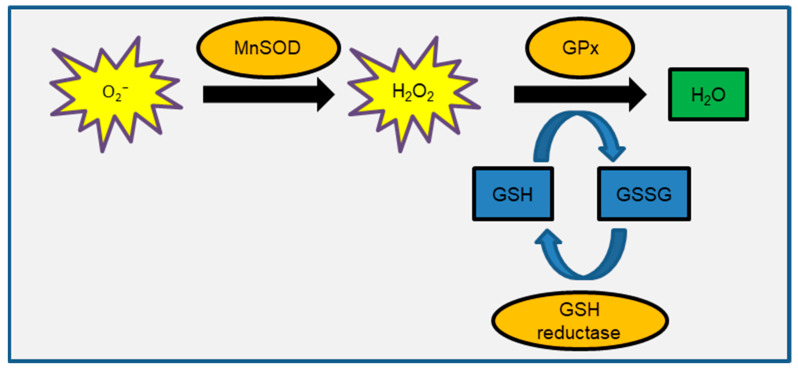
Schematic representation of GSH-mediated redox control.

Excessive ionizing radiation (IR) exposure is hazardous to human health because it causes tissue or organ function loss due to cell death and cellular senescence. Furthermore, radiation-induced genetic damage contributes significantly to the radiation carcinogenesis process. Radiation defense mechanisms in cells include cell cycle checkpoints, DNA repair, and cell death [1,2]. DNA double-strand breaks (DSBs) have been extensively studied in radiation biology research because nuclear DNA is thought to be the biological target of radiation. IR effects have been observed in cellular organelles other than the nucleus, such as the plasma membrane, cytoskeleton, mitochondria, endoplasmic reticulum, Golgi apparatus, and lysosome [3,4,5,6]. As an example of the scientific basis for IR effects on these cytoplasmic organelles, the bystander effect has been reported in the case of cytoplasmic irradiation of alpha particles using microbeam irradiation [7,8]. Mitochondria, like nuclei, contain their own genetic material and are thought of being IR targets [6,9,10]. Another paper discusses the role of mitochondria in radiation response [6]. Mitochondrial DNA (mtDNA) is devoid of a nucleosome structure containing histone proteins. Furthermore, due to the lack of some DNA repair machinery, the efficiency of mitochondrial DNA repair is lower than that of nuclear DNA. Because radiation-induced oxidized DNA, 8-hydroxydeoxyguanosine (8-OHdG), is more abundant in mtDNA than in nuclear DNA [11], the IR effects of mtDNA are expected to be stronger than those of nuclear DNA. Mitochondria are organelles that store cellular energy. Mitochondrial oxidative phosphorylation (OXPHOS) consumes oxygen and transfers electrons via substrate oxidation, resulting in an electrochemical gradient of protons (H^+^) between the mitochondrial membrane’s inner and outer sides. This mitochondrial membrane potential (ΔΨm) is used to generate adenosine triphosphate (ATP) as cellular energy [12]. Mitochondria are the primary source of reactive oxygen species (ROS) in the cell. During the electron transport chain reaction, superoxide (O_2_^−^) is produced by electron leak from complexes I and III [10,13,14]. As a result, ROS are produced in mitochondria. ROS are also produced after radiation exposure by radiolysis of water and endogenously during energy demand reactions in the mitochondria of eukaryotic cells. Because ROS are highly reactive and have a short lifecycle, it is thought that IR-induced delayed ROS production is caused by mitochondrial OXPHOS [15]. ROS are signaling pathway transmitters that also play physiological roles in cell proliferation. As a result, glutathione (GSH)-mediated redox control keeps ROS levels constant (Figure 1). Manganese superoxide dismutase (MnSOD) is an enzyme that converts O_2_^−^ to hydrogen peroxide (H_2_O_2_). Glutathione peroxidase (GPx) converts glutathione disulfide (GSSG) and water using H_2_O_2_ and GSH as substrates [16,17]. Nicotine amide adenine dinucleotide reduced form (NADPH)-dependent GSH reductase works to reduce GSSG in order to recover GSH. The GSH/GSSG ratio is used for assessment as a marker for oxidative stress. The MnSOD and nuclear factor κ-light-chain-enhancer of activated B-cells (NF-kappaB) cell signaling pathways are also involved in the radiation adaptation response and bystander effects seen with low-dose radiation [18,19,20]. MnSOD scavenges ROS to protect mitochondria from oxidative stress and is involved in tumor prevention. However, mitochondrial dysfunction and redox deregulation cause an increase in ROS levels, which leads to oxidative stress by oxidizing nuclei, proteins, lipids, and other substances [21]. As a result of O_2_^−^ leakage during the energy production process, mitochondria become toxic rather than detoxifying oxygen [16]. The effect of radiation on redox control was studied in normal human fibroblasts. We recently discovered that IR increased ROS levels by inhibiting GPx’s ability to scavenge ROS [22]. As a result, the GSH redox potential is critical in maintaining redox homeostasis after radiation. Mitochondria are vulnerable to ROS attack under these conditions [23]. IR damages the respiratory chain and causes mtDNA mutations. Abnormal mitochondrial metabolism increases the number of ROS produced during the OXPHOS process, causing oxidative damage to mitochondria yet again, resulting in a vicious cycle of mitochondrial oxidative stress. Perturbation of cellular redox control causes oxidative stress-related human diseases such as cancer, neurodegenerative diseases, and cardiac diseases [16,24,25,26,27].

In this review, the role of mitochondrial signaling in radiation carcinogenesis is discussed and our recent findings on mitochondrial signaling in response to DNA damage are highlighted.

## 2. Mitochondria-Mediated Various Signaling Pathways and Carcinogenesis

### 2.1. Nucleus to Mitochondria Signaling

**Figure 2:**
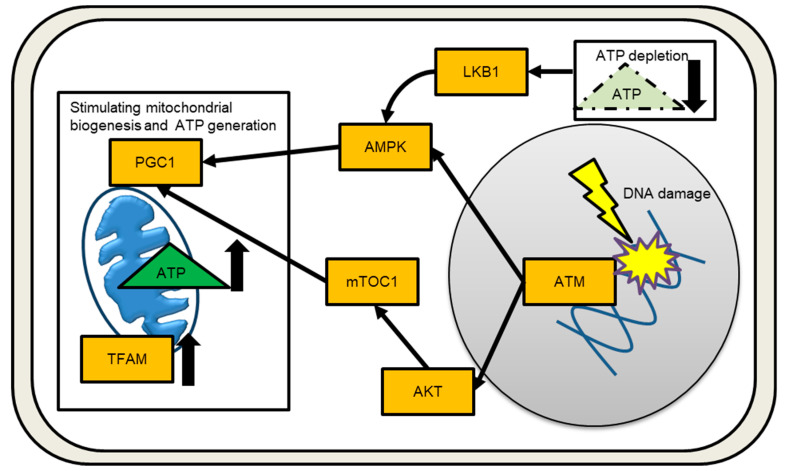
Signaling from the nucleus to the mitochondria in response to DNA damage is depicted schematically. Increased and decreased the amounts of ATP or TFAM are indicated by an arrow.

Processing stalled replication forks under replicative stress conditions can result in DSB [28], as can exogenous exposure to DNA-damaging agents such as IR, UV light, and anticancer drugs. DSBs immediately activate multiple molecular signaling pathways in order to carry out the DNA damage response (DDR) and maintain genomic stability. The trimeric MRE11-RAD50-NBS1 (MRN) complex recognizes, processes, and protects DNA ends [29]. Ataxia telangiectasia (AT) is an autosomal recessive disorder caused by the inactivation of the DNA damage sensor kinase ataxia telangiectasia mutated (ATM). The MRN complex recruits and aids in the activation of ATMs. ATM is the master DSB sensor, and the DNA damage signal spreads to other intracellular organelles, activating downstream effectors [30,31]. ATM recognizes IR-induced DNA DSBs via auto-phosphorylation [32]. The histone variant H2AX, which serves as a landmark for DNA repair proteins and regulates DNA repair, cell cycle progression, and cell death, is then phosphorylated by ATM [33,34,35,36,37]. Figure 2 depicts the damaged nucleus to mitochondria signaling. DNA damage signals are transported to mitochondria by the adenosine monophosphate (AMP)-activated protein kinase (AMPK) [38,39]. ATM-mediated AMPK signaling is currently being investigated because ATM has been shown not to directly phosphorylate AMPK [40]. The ATM/AKT/mammalian target of rapamycin (mTOR) pathway is another route to enhance mitochondrial biogenesis via transcriptional activation of PGC1 (Figure 2) [41]. The mitochondria contribute significantly to DDR by producing cellular energy in the form of ATP via OXPHOS. ATP appears to be required for DNA repair and chromatin remodeling [42]. When intracellular ATP is depleted and AMP levels rise, AMPK acts as an energy sensor, coordinating metabolic pathways [43]. When the internal ATP/ADP ratio falls, the AMPK/mTOR pathway suppresses cell growth [44]. AMPK is activated via Thr-172 phosphorylation in response to cellular energy depletions, such as nutrient deprivation, hypoxia, and mitochondrial respiration inhibition. LKB1 and Ca^2+^/calmodulin-dependent protein kinase (CaMKK) have been identified as AMPK upstream kinases [45,46]. Activated AMPK inhibits ATP-consuming pathways such as lipid, carbohydrate, and protein synthesis, while encouraging ATP production including mitochondrial biogenesis. AMPK activates the transcription factor nuclear respiratory factors 1 and 2 (NRF-1 and -2), as well as transcription factor A (TFAM), to activate transcription of nuclear-encoded mitochondrial genes [47,48,49]. IR increases mitochondrial mass and mtDNA copy number, which helps to meet the increased energy demands of DDR [50,51]. Radiation-induced bystander factors also increase mitochondrial mass [52]. Cells in the G2/M phase contained more mitochondrial than cells in the G1 or S phases. The presence of more mitochondria is linked to IR-induced G2/M cell cycle arrest [53].

### 2.2. Mitochondria as Regulators of Signal Transduction

Mitochondria are double-membrane structures found in the cytoplasm of eukaryotic cells. Each eukaryotic cell may contain hundreds of mitochondria, with each mitochondrion containing 2–10 copies of the mtDNA. Apart from nuclei, maternally inherited mitochondria are thought to be descendants of ancient bacteria and control their replication, transcription, and protein translation independently. The mitochondrial protein encoded by mammalian mtDNA is required for OXPHOS and the expression of mitochondrial genes such as transfer RNAs and ribosomal RNAs. To ensure survival and adaptation, mitochondrial functions include energy sensor and cellular defense systems against oxidative stress. Mitochondria have evolved signaling functions to communicate with the rest of the cell in order to adapt to toxic environmental insults. Cells do not initiate biological responses before integrating mitochondrial regulatory inputs. The ability of the mitochondria, for example, influences metabolic pathway choices in response to the cell’s energy demand. In the physiological response to hypoxia, mitochondria serve as the oxygen sensor. The generation of mitochondrial ROS leads to the activation of the transcriptional factor NRF2, which results in the expression of antioxidant and anti-inflammatory proteins. As a result, the release of second messengers from mitochondria causes a protective antioxidant response, which provides health benefits both in the short and long term. Morphological changes to mitochondria trigger nuclear DDR and DNA repair as retrograde signals [54]. On the contrary, mitochondrial components released from dysfunctional mitochondria trigger stress responses.

### 2.3. Mitochondrial ROS Signaling in Bystander Response

**Figure 3:**
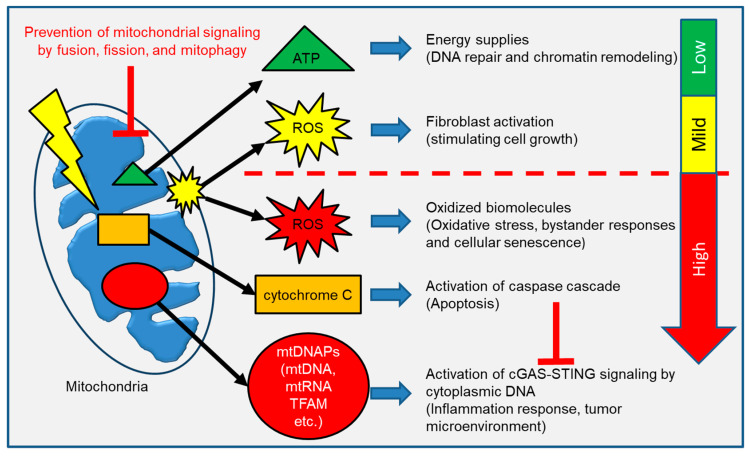
Mitochondrial components are released into the cytoplasm to communicate with the rest of the cells. ATP and ROS act as a second messenger molecule in a variety of physiologic responses. However, release of excess ROS, cytochrome C and mtDNAPs initiate a stress response against mitochondrial damage.

Various mitochondrial signaling pathways are depicted in Figure 3. It is widely accepted that IR causes bystander effects in cells that are indirectly traversed by a radiation track. It has been proposed that mitochondrial-derived ROS, such as hydroxyl radicals (OH^−^) and O_2_^−^, act as bystander signals in the radiation-induced bystander effect (RIBE) [55,56]. Thus, the delayed production of mitochondrial ROS mediates the long-term effects of IR in neighbor non-irradiated cells [23]. Mitochondria play an important role in the early stages of RIBE as well as the release of ROS generated during OXPHOS. The primary cause of oxidative stress is increased levels of ROS. Reactive nitrogen species (RNS), particularly NO, have been implicated in Nuclear Factor-κB–mediated signaling and are thought to contribute to RIBE [20,57]. RNAs, miRNAs, DNA, and cell transfers are all found in exosome-like vesicles (ELV). In RIBE, irradiated cells’ mtDNA migrates to non-irradiated cells, “spreading” the oxidative stress signal across a population of cells [58].

### 2.4. Mitochondria-Mediated Apoptosis

Apoptosis is caused by IR-induced severe DNA damage. It acts as an anticancer agent by killing tumor cells. To prevent tumor development, the tumor suppressor p53 is involved in numerous signaling pathways and induces apoptosis [59]. Neuronal apoptosis, on the other hand, has been linked to neurodegenerative diseases by loss of tissue function [60]. DDR, including the activation of mitochondrial-mediated apoptosis signaling, plays an important role in maintaining the integrity of genetic information by eliminating abnormal cells. Apoptosis is induced by mitochondrial outer membrane permeabilization (MOMP), which is induced by the pro-apoptotic effectors BAX and BAK [61]. In conjunction with apoptotic protease activating factor (Apaf), mitochondrial signaling, including the release of cytochrome C into the cytosol, promotes caspase cascade activation including effector caspases-3 and -7 [62]. Caspases are proteases that can cleave a variety of proteins. Caspase-activated DNase (CAD) is a caspase substrate involved in the cleavage of chromosomal DNA during apoptosis. Other cell death pathways, such as necrosis, pyroptosis, and ferroptosis, involve mitochondrial function in addition to apoptotic cell death [63,64]. Caspase-independent pathways control necrosis. During the early stages of necrosis, opening permeability transition pores disrupt mitochondrial plasma membrane integrity [65].

### 2.5. Role of Mitochondria on Cellular Senescence

Exogenous and endogenous stress and damage cause irreversible cell cycle arrest, which is a hallmark of cellular senescence [66]. Senescent cells limit cell proliferation’s replicative capacity via the p53/p21 and p16/pRb pathways, which halt cell cycle progression [66]. Tissue regeneration allows for the repair or replacement of damaged tissue through the renewal and growth of stem cells and progenitor cells. Senescent cells with a senescence-associated secretory phenotype, on the other hand, secrete inflammatory cytokines, growth factors, and proteases that can prevent neighboring cells from growing. The accumulation of senescent cells with age is linked to the promotion of aging and a number of age-related diseases [67]. Mitochondrial damage, excessive mitochondrial ROS, and DDR all occur sequentially, forming a mitochondrial ROS-mediated positive feedback loop that disrupts mitochondrial homeostasis. According to the free radical theory of aging, free radical damage is the primary driving force behind the aging process [68]. Aside from ROS, metabolic signaling pathways play a role in the induction of cellular senescence [69]. Mitochondrial dysfunction has been linked to the promotion of cellular senescence. Abnormal mitochondrial signatures and mitochondrial dysfunction are associated with age-related disease.

### 2.6. Mitochondrial Signaling in Inflammation

Mitochondria act as a master regulator of the metabolic switch in immune cell activation and immune response. A metabolic shift toward glycolysis and loss of OXPHOS capacity causes the activation of T cells, macrophages, and dendritic cells [70,71,72]. Instead of OXPHOS, activated macrophages use oxygen to produce NADPH oxidase-mediated ROS that are responsible for bacterial killing [73]. Under physiological conditions, mitochondrial-derived molecules play an important role as second messengers. In contrast, mitochondrial damage-associated molecular patterns (mtDAMPs) are endogenous danger signals produced by a variety of components that are normally sequestered in the mitochondria. Invading pathogens are defended against by innate immune responses in mammalian cells. Mitochondrial signaling, by acting as an initial signal, promotes immune responses. Mitochondrial dysfunction and increased ROS production result from mitochondrial injury. MOMP causes inflammatory signaling to be activated. Damaged cells release mitochondrial-derived intracellular molecules, such as mtDAMPs, into the cytoplasm or extracellular environment to initiate a pro-inflammatory response [74,75]. Immune systems recognize unmethylated CpG motifs in mtDNA as bacteria DNA. The cytosolic DNA sensor cyclic GMP-AMP synthase (cGAS) recognizes mtDNA in the cytoplasm and generates the second messenger 2′,3′-GMP-AMP (cGAMP), which activates the adaptor stimulator of interferon genes (STING)-mediated type interferon (IFN) response [76]. mtDNA is released into the cytosol when the mitochondrial outer membrane is permeabilized during apoptosis [64]. Meanwhile, caspase activation inhibited the activation of the mtDNA-mediated immune response activation [76]. Pathogen recognition receptors (PRRs) and pathogen-associated molecular patterns (PAMPs) are activated when mtDAMPs are released as a result of tissue injury. The presence of mtDAMPs has been linked to a number of diseases, including infection, asthma, ischemic heart disease, and cancer [77]. Measuring circulating mtDAMPS levels in patients may be useful as a biomarker for predicting disease severity and prognosis [77]. Indeed, mtDNA levels in plasma have been linked to patient mortality in medical intensive care units [78].

### 2.7. Mitochondrial Quality Control by Fusion, Fission, and Mitophagy

When mitochondria are damaged, cells have a mitochondrial homeostasis maintenance system that prevents mitochondrial components from leaking into the cytosol or extracellular matrix. There are thousands of mitochondria in somatic cells. The dynamic morphological change of fission and fusion events excludes damaged mitochondria and restores healthy mitochondria [79,80]. The fusion of mitochondria allows for the complementation of mitochondrial defects with mutant DNA by mixing mitochondria with wild-type DNA. The fusion proteins mitofusin 1 (Mfn1), Mfn2, and optic atrophy 1 (OPA1) are dynamin-related GTPases that are responsible for mitochondrial fusion in mammals. Drp1 (dynamin-related protein 1), on the other hand, mediates mitochondrial fission. Drp1 is phosphorylated [81] and recruited to the outer mitochondrial membrane to interact with mitochondrial receptor proteins, resulting in the division of a mitochondrion into two distinct organelles. Mitochondrial fission also helps with quality control. The fusion of healthy mitochondria with damaged mitochondria induced by IR causes significant changes in mitochondrial morphology for maintaining mitochondrial quality control [82,83,84]. In rat neurons, mitochondrial fusion was found to protect against low-dose IR [85]. IR induces Drp1-mediated mitochondrial fission in normal human fibroblasts [15]. On the other hand, mitophagy is the selective removal of damaged and dysfunctional mitochondria while leaving healthy mitochondria alone [86]. The loss of mitochondrial ΔΨm is primarily a sign of mitophagy in mammalian cells. The phosphoinositide 3-kinase (PI3K)–phosphatase with tensin homology (PTEN)-induced kinase 1 (PINK1) is normally unstable, but it becomes stabilized on the dysfunctional mitochondrial surface, where it recruits the E3 ubiquitin ligase Parkin. PINK1 phosphorylates both ubiquitin and Parkin at serine 65, increasing Parkin’s E3 activity on damaged mitochondria [87,88,89]. Parkin then promotes mitochondria degradation to maintain mitochondrial quality [90,91]. ATM promotes mitophagy activation by regulating the Pink1–Parkin pathway [92]. Meanwhile, PINK1 or Parkin deficiency impairs mitophagy’s ability to clear damaged mitochondria, resulting in mtDNA-mediated activation of inflammatory responses via cGAS-STING signaling [93].

### 2.8. Mitochondrial Dysfunction and Carcinogenesis

Mitochondrial dysfunction has been linked to a variety of diseases, including metabolic diseases, neurodegenerative diseases, and cancer [16,94,95,96]. Defects in mitochondrial function alter the cellular metabolism of cancer cells [97]. To achieve their high proliferation rate, cancer cells consume a much higher amount of glucose via glycolysis. Mitochondrial ROS play a role in tumor development through a variety of mechanisms, including oxidative stress, tumor cell proliferation, and chronic inflammation [98,99]. High ROS levels in cancer cells have been suggested as a cause of mitochondrial dysfunction [100]. ROS are genotoxic agents that can promote the initiation and progression process of multistage carcinogenesis [101]. These findings suggest a link between mitochondria-mediated oxidative stress and carcinogenesis [6,102]. Mutations in mtDNA have been discovered in a variety of human cancer cells [103,104,105]. Impaired mitochondrial functions such as ATP production, metabolism, calcium homeostasis, and apoptotic regulation promote tumor development [106]. Because of mitochondrial dysfunction (Warburg effect), cancer cells, unlike normal cells, use aerobic glycolysis for glucose metabolism [107]. In the absence of mitochondrial respiration defects, changes in energy metabolism caused by mitochondrial dysfunction allow cancer cells to acquire and tolerate proliferative potential. This modification is thought to be a tumor development marker [108]. Mitochondrial ROS enter the nucleus and damage nuclear DNA, contributing to genomic instability [109,110]. Because of genetic changes in nuclear DNA, cells gain a proliferative advantage.

Because it provides a nurturing environment for the malignant process, the tumor microenvironment plays critical roles in cancer development and progression. In addition to cancer cells, the tumor microenvironment includes stromal cells such as myofibroblasts and/or cancer-associated fibroblasts (CAFs), vascular endothelial cells, and tumor-associated immune cells. Fibroblasts mechanically support tissues by interacting between cells and remodeling excess extracellular matrix (ECM). CAFs are a major cellular component of tumor stroma with distinct properties from their normal counterparts. CAFs express α-smooth muscle actin (α-SMA), which is used to identify CAFs [111]. ROS-mediated mitochondrial signaling is thought to play a role in tumor microenvironment formations, which contribute to tumor development [112]. We recently discovered that IR-induced mitochondrial ROS activate transforming growth factor-β (TGF-β) signaling, which stimulates fibroblast activation via induction of α-SMA protein expression [113]. Fibroblast activation is used to repair and regenerate connective tissue after radiation injury. In an acute cellular immune response, activated fibroblasts modulate ECM production and accumulate lymphocytes, macrophages, and dendritic cells through the secretion of various growth factors, cytokines, and chemokines. When the healing process is complete, the activated fibroblasts vanish and return to their dormant state. However, persistent pro-fibrotic and pro-inflammatory cytokine and chemokine production by activated fibroblasts prevents the transition from an innate immune response to an acquired immune response, resulting in chronic inflammation and fibrosis [7]. DNA damage leads to incomplete wound healing by the induction of chronic inflammation and cellular senescence, which have been associated with fibrosis [114]. Radiation-induced fibrosis is a serious side effect of radiation treatment. We previously reported that activated fibroblasts with α-SMA expression survived following high doses of IR with >5 Gy for at least 24 h. Surprisingly, these activated fibroblasts appeared by exposure to low-doses with >0.1 Gy in the growth-restricted conditions in which cells were cultured in 0.5% or 0% fetal calf serum (FCS) [115]. Thus, when fibroblast cell growth is inhibited due to severe DNA damage or insufficient growth factor, activated fibroblasts may be retained for an extended period of time after irradiation. The interaction between IR-activated fibroblasts and malignant cancer cells promotes the growth and invasion of cancer cells through the release of paracrine growth factors [113]. Activated fibroblasts, for example, produce a variety of growth factors such as basic fibroblast growth factor (bFGF), TGF-β, and vascular endothelial growth factor (VEGF). Tumor cells, on the other hand, retain CAF properties that promote angiogenesis, ECM remodeling, and metastasis. Mitochondrial oxidative stress may be important in the radiation-induced carcinogenic process by promoting the formation of CAF, which is a cancer tissue [116,117,118]. Mitochondrial ROS are thought to influence tumor microenvironment formation and to promote cancer cell growth [113]. As a result, mitochondrial dysfunction contributes to the development of radiation-induced cancer [109].

### 2.9. Mitochondrial Signaling in Resistance to Cancer Treatment

Radioresistance of cancer cells remains a major limitation for radiotherapy. Alterations in mitochondrial function and metabolism confer tumor radioresistance [119,120,121]. Tumor immune escape is the barrier in cancer therapy. Programmed death ligand-1 (PD-L1), an immune checkpoint molecule, has a critical role in immune self-tolerance of cancer cells by binding to its receptor, programmed cell death protein-1 (PD-1), on T cells [122]. DNA damaging agents trigger DDR, which leads to upregulation of PD-L1 expression in cancer cells [123]. PD-L1 promotes increases in the mRNA stability of DNA repair proteins, so that cancer cells are resistant to DNA damage [124]. Together with PD-L1, TGFβ is thought to be a target molecule for overcoming the immune evasion of tumor cells [125]. PD-L1/PD-1 blockade by AMPK activation significantly enhances cancer immunotherapy [126,127]. Pink1 regulates mitochondrial localization of PD-L1 for its degradation via mitophagy [128].

### 2.10. Human Cancer Risks Attributable to Radiation

Radiation cancer risks are a major public health concern. The Life Span Study cohort’s epidemiological data among Hiroshima and Nagasaki atomic bomb survivors is the most reliable scientific evidence in radiation risk assessment for radiological protection. This cohort’s large sample size, which includes a wide range of ages and individual radiation exposure doses, has greater statistical power than other smaller radiation studies. Excess radiation risk of leukemia appeared ~2 years after the bombing and peaked 6–7 years later [129,130]. A surplus of solid cancers, on the other hand, became apparent a few decades later [131]. Throughout life, the incidences of leukemia and solid cancer increase linearly in proportion to the radiation dose. Radiation-induced cancer is classified as a stochastic effect with no dose threshold level based on the absorbed dose, age at exposure, and gender. There is concern about radiation exposure among people causing physical and mental health issues in the Fukushima nuclear accident, which occurred on 11 March 2011. Chronic physical diseases such as depression and alcoholism have been reported in Fukushima nuclear accident victims [132,133]. As a result, the health risks associated with low-dose radiation (below ~100 mSv) must be clarified. Basic science studies and radiation risk knowledge acquisition can help to deepen the general public’s understanding of radiation.

### 2.11. Radiation Protection by Targeting Mitochondria

Acute radiation syndrome (ARS) is caused by a short period of whole-body or significant partial-body irradiation of more than 0.5 Gy [134]. It is widely accepted that rapidly dividing hematopoietic stem cells and bone marrow progenitor cells are extremely sensitive to IR. Excessive 0.5 Gy exposure resulted in a significant decrease in the number of peripheral blood cells. Lymphocytes and monocytes are quickly cleared from the bloodstream at first. The granulocyte and platelet count then decreases over time. Red blood cells have a slower clearance rate over a longer time period. Clinical symptoms, such as hematopoietic and gastrointestinal sub-syndromes, appear within the first two months of exposure [135]. To improve care for radiation victims in a radiation emergency, the development of appropriate radiation protective agents is urgently needed. Antioxidants, nutrients, and phytochemicals are candidates for use as radiation protective agents in a variety of radiation exposure scenarios [136]. For example, potassium iodide is used as a thyroid blocking agent in the presence of I-131 radioiodine [137]. The ability of cysteine to scavenge free radicals plays a role in radiation protection from whole-body irradiated animals [138]. By mitigating the hematopoietic sub-syndrome of ARS, granulocyte colony-stimulating factor (G-CSF) induces hematopoiesis and improves mouse survival from lethal total-body gamma-irradiation [139]. Radioprotective agents have thus far been developed by providing pre-administration treatment for radiation workers or cancer patients who are likely to be exposed to radiation [140]. However, in nuclear or radiological accidents, unforeseeable consequences of radiation exposure may occur among large populations. As a result, radioprotective agents should be developed under different radiation exposure scenarios, including post-radiation treatment. Serious side effects of radiation-protective agents are major issues in clinical trials [136]. It is truly important to translate scientific findings from animal studies into practical clinical applications. Human health effects of radiation include both acute radiation injury clinical symptoms and late-onset radiation carcinogenesis [141]. In the case of acute radiation injury, mitochondrial signaling is linked to tissue and organ function loss via induction of apoptotic cell death. Furthermore, radiation-related carcinogenesis is linked to impaired mitochondrial function and mitochondria-mediated oxidative stress. Therefore, mitochondria play an important role in radiation responses in both acute and late effects of carcinogenesis. It is critical to protect mitochondria in order to mitigate radiation effects throughout life. A new radioprotective agent that targets mitochondria was recently investigated [142]. In irradiated normal human fibroblasts, IR has been shown to prevent GSH-mediated ROS scavenging by inactivation of GPx, resulting in damage to mitochondria, mitochondrial dysfunction, metabolic oxidative stress, and prolonged cell injury [22]. N-acetyl-5-methoxy-tryptamine (Melatonin) and MitoEbselen-2, a mitochondria-targeted GPx mimic, were used to stimulate GPx, which maintains mitochondrial-derived ROS levels. Some ROS indicators include 2′,7′-dichlorofluorescin diacetate (DCFDA: intracellular ROS levels), MitoSOX-red (mitochondrial-delivered ROS), and OxiORANGE (hydroxyl radicals). Even after radiation exposure, melatonin or MitoEbselen-2 can maintain GPx activity and intracellular ROS levels. Pre- and post-radiation treatment with melatonin or MitoEbselen-2 suppressed radiation responses such as γ-H2AX, Nrf2, Tom20 (mitochondria marker), parkin, and α-SMA. As a result, both drugs are effective in mitigating radiation-induced DSB, mitochondrial damage, and fibroblast activation. Concurrent administration of melatonin or mitoEbselen-2 with radiation resulted in no metabolic oxidative stress or mitochondrial injury. These drugs are potentially effective countermeasures for mitochondria-mediated oxidative stress.

## 3. Conclusions

Mitochondrial-to-nuclear communication regulates a variety of biological responses such as metabolism, oxidative stress response, bystander response, apoptosis, and inflammation response. Mitochondria serve as a signaling platform by the release of mitochondrial components to the cytoplasm and ECM to modulate cellular communication. Mitochondrial signaling regulates not only physiological responses but also causes the detrimental effects of oxidative damage. Mitochondrial dysfunction promotes the initiation of radiation-related tumors through a variety of signaling pathways. Mitochondrial-targeted agents can mitigate mitochondrial-mediated tumor microenvironment formation by suppressing the appearance of activated fibroblasts after radiation A better understanding of the underlying mechanisms will hopefully lead to a better understanding of radiation risks. Understanding how radiation affects mitochondria and their roles in radiation carcinogenesis is critical for understanding how radiation affects human health. More research into the role of mitochondrial stress signaling in the onset and progression of radiation carcinogenesis is needed in the future to better understand radiation risk.

## Data Availability

Not applicable.
